# Remote real-time supervision of prehospital point-of-care ultrasound: a feasibility study

**DOI:** 10.1186/s13049-021-00985-0

**Published:** 2022-03-24

**Authors:** Martina Hermann, Christina Hafner, Vincenz Scharner, Mojca Hribersek, Mathias Maleczek, Andreas Schmid, Eva Schaden, Harald Willschke, Thomas Hamp

**Affiliations:** 1 Department of Anaesthesia, General Intensive Care and Pain Medicine, Medical University of Vienna, Waehringer Guertel 18-20, 1090 Vienna, Austria; 2 Ludwig Boltzmann Institute for Digital Health and Patient Safety, Vienna, Austria

**Keywords:** Ultrasound, Ultrasonography, Sonography, Ultrasonics, Diagnostic imaging, Echocardiography, Emergency medicine, Patient safety, Telemedicine, Anaesthesiology

## Abstract

**Background:**

Although prehospital point-of-care ultrasound (POCUS) is gaining in importance, its rapid interpretation remains challenging in prehospital emergency situations. The technical development of remote real-time supervision potentially offers the possibility to support emergency medicine providers during prehospital emergency ultrasound. The aim of this study was to assess the feasibility of live data transmission and supervision of prehospital POCUS in an urban environment and so to improve patients’ safety.

**Methods:**

Emergency doctors with moderate ultrasound experience performed prehospital POCUS in emergency cases (n = 24) such as trauma, acute dyspnea or cardiac shock using the portable ultrasound device Lumify™. The ultrasound examination was remotely transmitted to an emergency ultrasound expert in the clinic for real-time supervision via a secure video and audio connection. Technical feasibility as well as quality of communication and live stream were analysed.

**Results:**

Prehospital POCUS with remote real-time supervision was successfully performed in 17 patients (71%). In 3 cases, the expert was not available on time and in 1 case remote data transmission was not possible due to connection problems. In 3 cases tele-supervision was restricted to video only and no verbal communication was possible via the device itself due to power saving mode of the tablet.

**Conclusion:**

Remote real-time supervision of prehospital POCUS in an urban environment is feasible most of the time with excellent image and communication quality.

*Trial registration:* ClinicalTrials Number NCT04612816.

**Supplementary Information:**

The online version contains supplementary material available at 10.1186/s13049-021-00985-0.

## Background

Point-of-care ultrasound (POCUS) plays an important role for physicians in decision making when treating critically ill patients. Due to the technical progress of portable ultrasound devices, POCUS is no longer reserved for the in-hospital setting, but is increasingly used in the field by prehospital emergency medicine providers across the world [1].

However, during emergency situations POCUS can remain challenging. Preclinical conditions (e.g. noise, access to the body in immobilized patients, cold weather, light and limited resources) can complicate the adequate execution and interpretation of POCUS [2]. Furthermore, rapid translation of ultrasound findings into meaningful therapeutic consequences is highly demanding and requires appropriate training [3]. However, if POCUS is correctly performed and the patients’ condition allows emergency medicine providers a careful ultrasound examination, it is cpossible to differentiate life-threatening diagnoses [4–7].

The development of tele-ultrasound as a branch of tele-medicine offers the opportunity of performing POCUS under the supervision of an expert who provides support in this challenging situation. Although several in-hospital studies demonstrated that tele-POCUS is feasible and beneficial for the patient, there is lack of evidence regarding the feasibility of live supervision of POCUS in the prehospital setting or on its impact on the outcome for patients [8–13].

This study aims to investigate the technical feasibility of tele-POCUS in a physician provided prehospital emergency medicine system, to identify obstacles to live data transmission and to improve diagnostic accuracy.

## Methods

### Study design

This study was designed as a feasibility trial of tele-POCUS in a physician provided emergency medical service in Vienna, Austria. Ethical approval (Number:1771/2020) was obtained by the Ethics Committee of the Medical University of Vienna (Martin Brunner, MD) before patient enrolment. The study conformed to the Declaration of Helsinki guidelines regarding research on human subjects and followed the tenets of Good Clinical Practice. The trial was registered before enrolment at ClinicalTrials.gov by the principal investigator Martina Hermann (10/2020 ClinTrials.gov NCT04612816). Between October 19, 2020 and May 27, 2021, 24 prehospital performed POCUS examinations during dayshifts, from 07.30 to 15.30 were live transmitted (audio and video) to an expert located at the Medical University of Vienna. Subsequent to the rescue mission, the physician evaluated the feasibility and quality of POCUS and reported technical problems using a questionnaire and to improve patients’ safety and diagnostic assurance.

### Emergency physicians

The participating rescue physicians (n = 4) were residents and specialists at the Department of Anaesthesia, General Intensive Care and Pain Management of the Medical University of Vienna with clinical experience of a least 3 years and moderate experience in in-hospital POCUS (daily clinical routine examinations at intensive care units, in the perioperative setting in anaesthesia and the echocardiography simulator).

The supervisor was a specialist in anaesthesia and critical care medicine at the Medical University of Vienna with the European Diploma in advanced critical care echocardiography.

### Patients

Patients treated by the participating emergency physicians were included, if prehospital POCUS was performed due to at least one of the following criteria: trauma, acute dyspnea or circulatory failure. Informed consent was obtained post hoc. CONSORT diagram is available Additional file 1.

### Prehospital ultrasound and data transmission

**Fig. 1:**
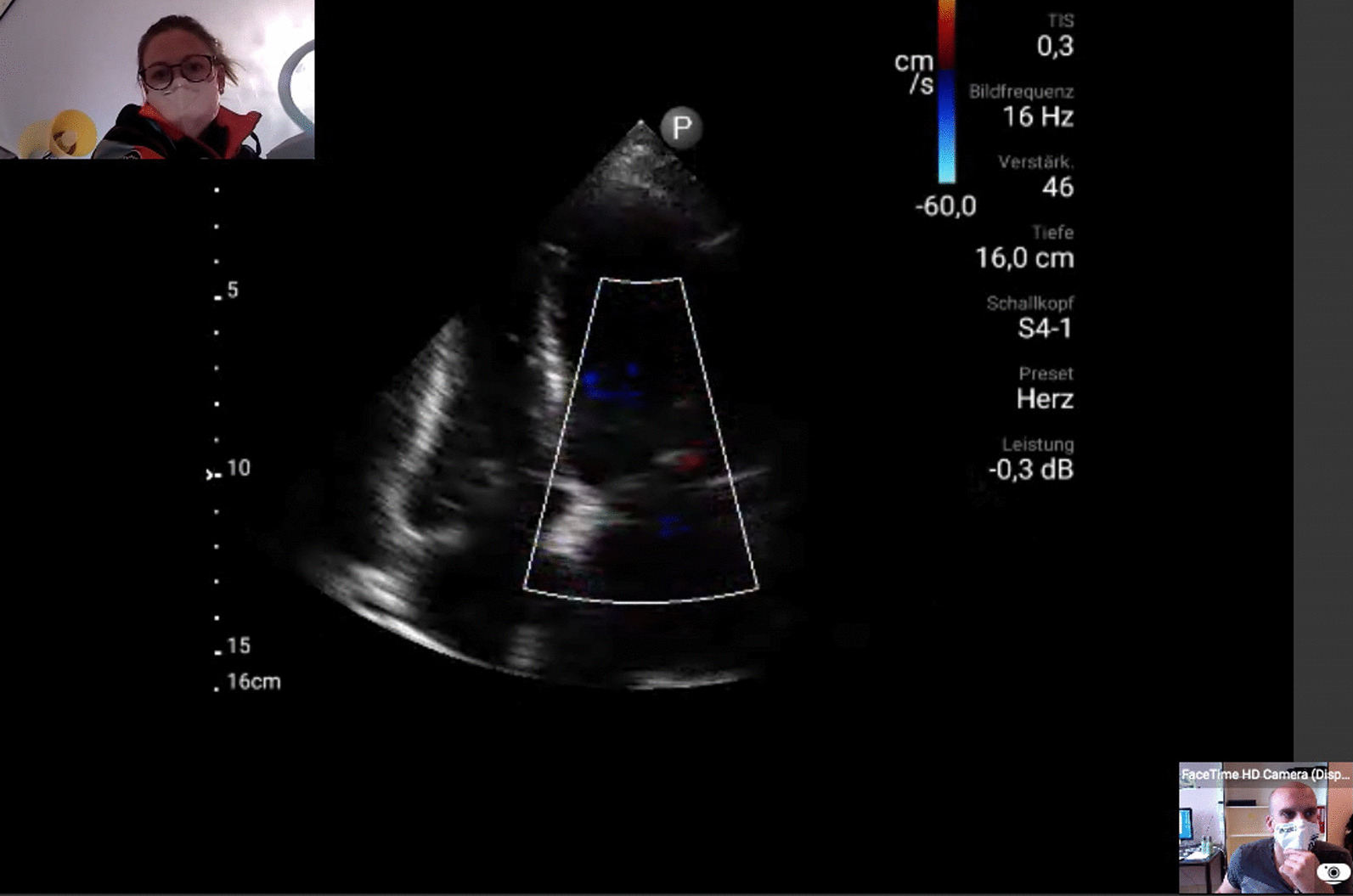
Supervisor’s view: ultrasound image, prehospital emergency doctor left corner, supervisor right corner

POCUS was performed with the portable ultrasound device Lumify™ (Philips Ultrasound, Inc., 22,100 Bothell-Everett Hwy Bothell, WA 98021-8431 USA). For transthoracic echocardiography, the transducer S4-1 (4–1 mHz) and for transabdominal sonography, the transducer C5-2 (5–2 mHz) were used. POCUS was performed on-scene according to standardized protocols of emergency ultrasound (e.g. Focus-assessed transthoracic echocardiography, FATE; Extended Focused Assessment with Sonography for Trauma, eFAST; Rapid Ultrasound for Shock and Hypotension, RUSH). The examination was chosen according to the leading clinical symptom (e.g. dyspnea, cardiac arrest). Concomitant to the start of POCUS, remote data transmission was initiated utilizing the interactive audio–video platform Reacts (Remote Education, Augmented Communication, Training and Supervision, Philips Ultrasound, Inc., 22100 Bothell-Everett Hwy Bothell, WA 98021-8431 USA), which offers secure data transfer and live communication with the expert (Fig. 1). To establish connection, a mobile 4G-SIM-card was used, which links to the strongest signal for a defined region regardless of the provider.

### Data collection

Data collection was performed by the emergency doctor at end of mission as well as by the remote supervisor concerning image quality and sonography findings. Demographic data of the patients (age, BMI, gender) as well as inclusion criteria for POCUS (trauma, acute dyspnea or circulatory failure) and additionally other symptoms and comorbidities were recorded. Image quality of POCUS as well as quality of communication and live stream were rated on a numeric scale (1 = excellent to 10 = poor). Ultrasound specific variables (performed scans, duration of POCUS and transmission), the availability of the expert on time, delay of POCUS due to problems with data transmission as well as the occurrence of technical problems were evaluated. The questionnaire is found as Additional file 2.

### Statistical analysis

Statistical analysis was performed using Prism 9.0 software (GraphPad, San Diego, CA, USA). Demographic data are presented as mean ± standard deviation (SD) or median (25–75th percentile). This study was designed to test the feasibility of this new method and no comparable trials have been reported yet. We therefore decided to set sample size to 25 patients which is a common sample size for feasibility studies and allows to get a rough estimate of the magnitude of the main outcome parameter [14].

## Results

### Baseline characteristics

Between October 19, 2020 and May 27, 2021, a total of 24 emergency patients were included in this trial. Baseline characteristics are depicted in Table 1. The leading symptom for performing prehospital POCUS was circulatory failure (14/24 patients, 58%) whereas trauma with acute dyspnoea was the reason for emergency ultrasound in one case only.

**Table 1:** Baseline characteristics

Age (years, SD)	69 (± 17)
Male (number, %)	14 (58%)
Female (number, %)	10 (42%)
BMI (kg/height m^2^, SD)	26 (± 4)
Comorbidities (number, %)	
Heart failure COPD/asthma/interstitial lung disease Coronary heart disease Diabetes mellitus Valvular heart disease Arterial hypertension Stroke	10 (42%) 7 (29%) 5 (21%) 5 (21%) 2 (8%) 2 (8%) 1 (4%)
Leading clinical symptom for ultrasound examination (number, %)	
Circulatory failure Acute dyspnoea Circulatory failure + acute dyspnoea Trauma + acute dyspnea	14 (58%) 5 (21%) 4 (17%) 1 (4%)

Data are presented as mean (± SD) and numbers (%)

**Table 2:** Performed ultrasound examinations

Performed ultrasound examinations	
TTE TTE + LUS eFAST	7 (29%) 16 (67%) 4 (17%)

TTE, transthoracic echocardiography; LUS, lung ultrasound; eFAST, extended focused assessment with sonography for trauma

Data are presented as total number (%)

**Table 3:** Reported technical problems

Technical problems (number, %)	
Tele-supervision not possible Expert not available No internet connection No sound (due to power saving mode)	7 (29%) 3 (13%) 1 (4%) 3 (13%)
Restricted tele-supervision Log-in disconnection Weak internet connection	5 (%) 3 (13%) 2 (8%)

Data are presented as total number (%)

### Ultrasound examination

Transthoracic echocardiography (TTE) was performed in 23 patients (96%) according to the FATE protocol including parasternal long axis in 15 patients (62%), parasternal short axis in 11 patients (46%), apical four-chamber cardiac view in 21 patients (88%) and subxiphoidal scan in 18 patients (75%). Lung ultrasound (LUS) was done in 17 patients (71%). Recessus hepatorenalis (Morison-Pouch) and Recessus splenorenalis (Koller-Pouch) were scanned in 4 patients (17%) according to the eFAST and RUSH protocol, while the bladder view was only included in 3 instances (13%). Median duration of POCUS was six minutes (IQR 4.0–8.0), during 66.7% (four minutes) of that time remote supervision was performed. The performed echocardiography scans primary depended on the suspected diagnosis. Solely ultrasound windows deemed relevant for diagnosis were applied (Tables 2).

### Remote real-time supervision

Remote real-time supervision was successfully performed during dayshifts in 17 of 24 cases (71%). In 3 cases the expert was not available on time and in 1 case remote data transmission was not possible due to connection problems. In 3 cases the power saving mode of the tablet resulted in real-time supervision without audio connection via the tablet, therefore the supervisor was called by a cell phone. Due to prolonged connection establishment of the remote real-time supervision, a delay in supervision of 20 s was reported in 3 cases. In 1 case weak internet connection was described (Table 3).

**Fig. 2:**
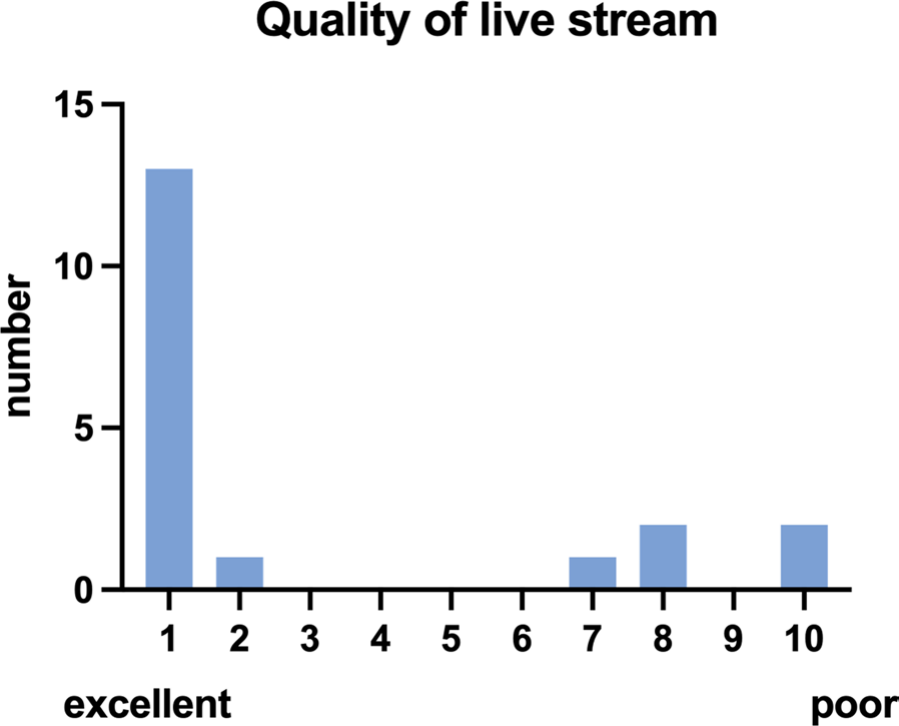
Quality of live stream: data are presented as total number

**Fig. 3:**
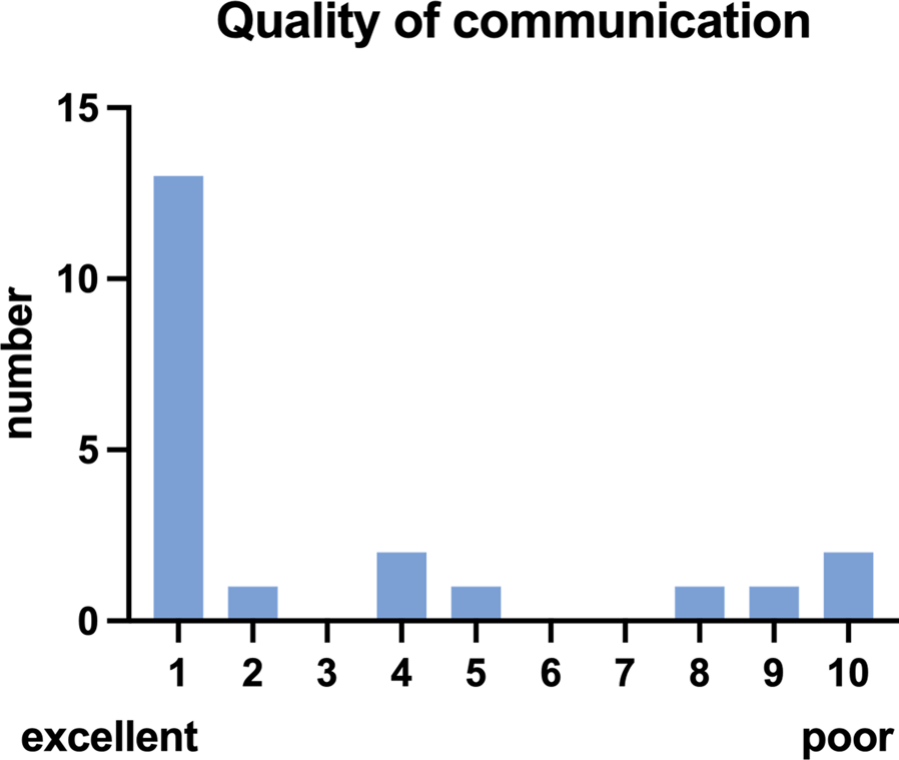
Quality of communication: data are presented as total umber

On average, image quality of live stream (Fig. 2) was rated with 1.0 (IQR 1.0–7.0) and quality of communication (Fig. 3) achieved a rating of 1.0 as well (IQR 1.0–4.5).

## Discussion

In preparation to further clinical studies this study was conceived, and its results demonstrate that remote real-time supervision of POCUS in a physician based prehospital emergency service is feasible with excellent image and communication quality as only in one case no internet connection was available due to a complex building architecture. Furthermore, the reported bug with the handling of the ultrasound device that was encountered thrice in the study and resulted in the absence of sound transmission concomitant to excellent video quality was that the tablet activated an energy-saving mode thereby cancelling sound transmission. In these cases, verbal communication needed to be established via separate cellphones. After the problem was recognized, participating emergency doctors were informed, and the technical trouble could be prevented.

The quality of communication and live stream of the investigated device rated as excellent offers the opportunity for further prehospital studies with focus on patient specific outcome parameters. As a few studies have demonstrated that tele-supervised physicians performed scans of better quality in in-hospital settings [2, 3, 5], a benefit may also be expected in prehospital emergency medicine.

While telemedicine is described as a concept for prehospital care [15], the effect of tele-supervision of prehospital POCUS has been explored sparely [1]. A few studies investigating feasibility and the effect of remote real-time supervision of ultrasound have been published, which mostly focus on novice ultrasound practitioners being mentored by sonography experts and attest an adequate image quality for diagnosis [16]. The majority of previous research focuses on either remote or rural regions with limited medical services, e.g. cruise ships [17], or medical staff with a very limited training in the interpretation of ultrasound imagery [18]. Boniface et al. demonstrated that paramedics with no prior ultrasound experience could perform eFAST under remote guidance of an experienced physician [1], while Eadie et al. reported on the successful application of eFAST and transcranial ultrasound by medical students with no prior sonography experience in 16 remote locations in Scotland with the aid of tele-supervision [19]. However, opinions on prehospital real-time remotely supervised sonography performed by novices to ultrasound differ strongly, especially by profession [20]. In comparison, the organizational structure of prehospital emergency medicine in Vienna, Austria, has emergency physicians with previous experience both in the performance and interpretation of sonography conducting POCUS in the prehospital setting rendering this controversy non-applicable.

Although POCUS gains in importance in emergency medicine, the indications for the prehospital setting remain unclear. Trauma and acute dyspnoea are among a limited number of clearly defined indications to balance rapidity with effectiveness [21]. In trauma patients, pre-hospital performed eFAST offers the possibility to identify severe thoracic and abdominal injuries before hospital admission [22, 23]. The detection of hidden bleeding can change the treatment strategy (e.g. fluid therapy, rapid transportation to a level one trauma center) [24]. As time plays an important factor in trauma, an earlier detection of a severe thoracic or abdominal injury may improve patient’s outcome [2, 25]. Several studies demonstrated that in patients suffering from acute dyspnoea lung ultrasound can be performed quickly and several pathologies (e.g., pleural effusions, pulmonary edema, pneumothorax) can be identified rapidly [5, 26]. Especially in a prehospital setting, lung ultrasound in combination with echocardiography can help to differentiate between cardiac and pulmonary causes of the very common symptom of acute dyspnea in emergency medicine [5, 7, 21, 27]. Real-time tele-supervision may support the prehospital emergency doctor, who is technically able to acquire adequate images, but lacks advanced image interpretation skills. Especially in ambiguous clinical scenarios, during time-critical situations, ultrasound supervision for emergency doctors might therefore improve patients’ safety and the choice of destination hospital. Even in urban regions with a high density of hospitals choosing the correct hospital initially is important, as inter-hospital transfer is time consuming. However, further studies are necessary to investigate the usefulness of real-time tele-supervision of emergency doctors with different levels of training and experience in POCUS and prehospital emergency medicine. Based on the feasibility of this study, further studies are already planned to investigate possible benefits of POCUS live transmission in the preclinical setting.

## Limitations

The trial was conducted in an urban environment with excellent internet and phone network coverage, leaving the transferability of the results to rural areas questionable.

Furthermore, the initial response times for emergency doctors and time required to transport patients to clinics are short with a high density of hospitals. The potential gain of preclinical ultrasound of earlier diagnosis and consequent differentiation in therapy and the choice of target hospital increases with the distances between emergency location and base and hospital respectively.

In a considerable number of ultrasound exams, it was not possible to acquire all images required for completion of the standardized POCUS protocol. This observation requires further investigation as performing an incomplete POCUS evaluation might be detrimental for patients. In addition, only one trauma patient was included in this feasibility trial and image acquisition was again limited due to immobilization of this patient. Further studies are therefore necessary to investigate the usefulness of real time remote tele-ultrasound supervision in this specific patient population and also the consequences of incomplete POCUS exams in the prehospital setting.

Our feasibility study demonstrated that an improvement in communication and organization is required to ensure that the clinical expert is available for supervision as remote tele-supervision was not possible in 3 cases due to the clinical workload of the expert. This aspect should be considered, when initiating tele-supervision for prehospital emergency doctors, while performing POCUS on emergency scene. Due to the feasibility character of this study only 4 emergency doctors and 1 expert performed POCUS during dayshifts. These factors explain the extended study duration of 8 months. 

However, based on the results of this feasibility trial, we have already planned additional larger studies that will investigate more clinically relevant questions in the area of respiratory respiratory failure and cardio pulmonary resuscitation. For additional information see https://clinicaltrials.gov/ct2/results?cond=live+stream+of+prehospital&draw=2&rank=4#rowId3.

## Conclusion

The present study adds evidence that remote real-time supervision of emergency physicians performing POCUS in prehospital settings in an urban area is technically feasible with excellent quality of communication and live stream most of the time, however the impact on the patient’s outcome remains to be elucidated.

## Supplementary Information

**Additional file 1:** Consort diagram

**Additional file 2:** Questionnaire on ultrasound and transmission quality

**Additional file 3:** Consort Checklist

## Data Availability

The datasets analyzed during the current study are available from the corresponding author on reasonable request. Exemplary ultrasound loops are available from the corresponding author on request.

## Abbreviations

eFAST: Extended Focused Assessment with Sonography for Trauma
FATE: Focus-assessed transthoracic echocardiography
LUS: Lung ultrasound
MUV: Medical University Vienna
POCUS: Point-of care ultrasound
REACTS: Remote Education, Augmented Communication, Training and Supervision
RUSH: Rapid Ultrasound for Shock and Hypotension
TTE: Transthoracic echocardiography

## References

[1] Boniface KS, Shokoohi H, Smith ER, Scantlebury K (2011). Tele-ultrasound and paramedics: real-time remote physician guidance of the Focused Assessment With Sonography for Trauma examination. Am J Emerg Med.

[2] Rudolph SS, Sorensen MK, Svane C, Hesselfeldt R, Steinmetz J (2014). Effect of prehospital ultrasound on clinical outcomes of non-trauma patients–a systematic review. Resuscitation.

[3] Moore CL, Copel JA (2011). Point-of-care ultrasonography. N Engl J Med.

[4] Weilbach C, Kobiella A, Ruschulte H (2015). Notfallsonographie im Rettungsdienst. Notfall + Rettungsmedizin.

[5] Laursen CB, Hanselmann A, Posth S, Mikkelsen S, Videbaek L, Berg H (2016). Prehospital lung ultrasound for the diagnosis of cardiogenic pulmonary oedema: a pilot study. Scand J Trauma Resusc Emerg Med.

[6] Breitkreutz R, Ilper H, Seeger FH (2008). Ultraschall für notfälle: anwendungen im rettungsdienst. Notfallmedizin Up2Date.

[7] Scharonow M, Weilbach C (2018). Prehospital point-of-care emergency ultrasound: a cohort study. Scand J Trauma Resusc Emerg Med.

[8] Weilbach C, Kobiella A, Rahe-Meyer N (2017). Introduction of prehospital emergency ultrasound into an emergency medical service area. Anaesthesist.

[9] Fevang E, Lockey D, Thompson J, Lossius HM, Torpo Research C (2011). The top five research priorities in physician-provided pre-hospital critical care: a consensus report from a European research collaboration. Scand J Trauma Resusc Emerg Med.

[10] Grant B, Morgan GJ, McCrossan BA, Crealey GE, Sands AJ, Craig B (2010). Remote diagnosis of congenital heart disease: the impact of telemedicine. Arch Dis Child.

[11] Jensen SH, Duvald I, Aagaard R, Primdahl SC, Petersen P, Kirkegaard H (2019). Remote real-time ultrasound supervision via commercially available and low-cost tele-ultrasound: a mixed methods study of the practical feasibility and users' acceptability in an emergency department. J Digit Imaging.

[12] Kim C, Kang BS, Choi HJ, Lim TH, Oh J, Chee Y (2015). Clinical application of real-time tele-ultrasonography in diagnosing pediatric acute appendicitis in the ED. Am J Emerg Med.

[13] Walcher F, Kirschning T, Muller MP, Byhahn C, Stier M, Russeler M (2010). Accuracy of prehospital focused abdominal sonography for trauma after a 1-day hands-on training course. Emerg Med J.

[14] Strnad M, Prosen G, Borovnik LV (2016). Bedside lung ultrasound for monitoring the effectiveness of prehospital treatment with continuous positive airway pressure in acute decompensated heart failure. Eur J Emerg Med.

[15] Amadi-Obi A, Gilligan P, Owens N, O'Donnell C (2014). Telemedicine in pre-hospital care: a review of telemedicine applications in the pre-hospital environment. Int J Emerg Med.

[16] Salerno A, Kuhn D, El Sibai R, Levine AR, McCurdy MT. Real-time remote tele-mentored echocardiography: a systematic review. Medicina (Kaunas) 2020;56:12. 10.3390/medicina56120668 PMC7761589 33276628

[17] Boniface KS, Sikka N, Page N, Peretz A, Shokoohi H (2020). A cruise ship emergency medical evacuation triggered by handheld ultrasound findings and directed by tele-ultrasound. Int Marit Health.

[18] Su MJ, Ma HM, Ko CI, Chiang WC, Yang CW, Chen SJ (2008). Application of tele-ultrasound in emergency medical services. Telemed J E Health.

[19] Eadie L, Mulhern J, Regan L, Mort A, Shannon H, Macaden A (2018). Remotely supported prehospital ultrasound: a feasibility study of real-time image transmission and expert guidance to aid diagnosis in remote and rural communities. J Telemed Telecare.

[20] Marsh-Feiley G, Eadie L, Wilson P (2018). Paramedic and physician perspectives on the potential use of remotely supported prehospital ultrasound. Rural Remote Health.

[21] Zanatta M, Benato P, De Battisti S (2018). Pre-hospital lung ultrasound for cardiac heart failure and COPD: is it worthwhile?. Crit Ultrasound J.

[22] Jørgensen H, Jensen CH, Dirks J (2010). Does prehospital ultrasound improve treatment of the trauma patient? A systematic review. Eur J Emerg Med.

[23] Walcher F, Weinlich M, Conrad G, Schweigkofler U, Breitkreutz R, Kirschning T (2006). Prehospital ultrasound imaging improves management of abdominal trauma. Br J Surg.

[24] Gomes E, Araujo R, Carneiro A, Dias C, Costa-Pereira A, Lecky FE (2010). The importance of pre-trauma centre treatment of life-threatening events on the mortality of patients transferred with severe trauma. Resuscitation.

[25] Botker MT, Jacobsen L, Rudolph SS, Knudsen L (2018). The role of point of care ultrasound in prehospital critical care: a systematic review. Scand J Trauma Resusc Emerg Med.

[26] Kristensen MS, Teoh WH, Graumann O, Laursen CB (2014). Ultrasonography for clinical decision-making and intervention in airway management: from the mouth to the lungs and pleurae. Insights Imaging.

[27] Pietersen PI, Mikkelsen S, Lassen AT, Helmerik S, Jorgensen G, Nadim G (2021). Quality of focused thoracic ultrasound performed by emergency medical technicians and paramedics in a prehospital setting: a feasibility study. Scand J Trauma Resusc Emerg Med.

