# Supplementary material for: Remote real-time supervision of prehospital point-of-care ultrasound: a feasibility study
Source: Scand J Trauma Resusc Emerg Med. 2022 Mar 24;30:23. doi: 10.1186/s13049-021-00985-0 (PMC8944068; doi:10.1186/s13049-021-00985-0)
Supplement: Supplementary file 1 — Additional file 1: Consort diagram [file 13049_2021_985_MOESM1_ESM.doc]

**
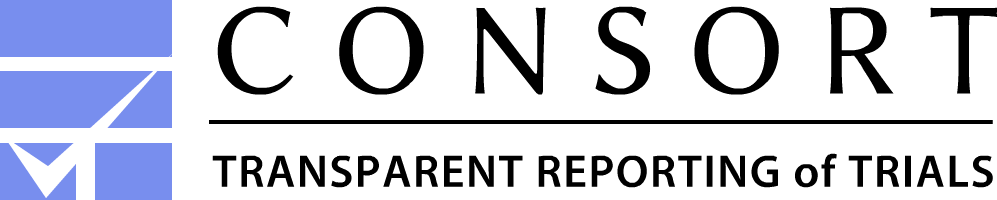
**

**CONSORT 2010 Flow Diagram**

**Analysis**

**Enrollment**

Assessed for eligibility (n=25)

Excluded (n=1)

♦  Declined to participate (n=1)

Analysed (n=24)

♦ Excluded from analysis (n=0)

No randomization
