# Supplementary material for: Remote real-time supervision of prehospital point-of-care ultrasound: a feasibility study
Source: Scand J Trauma Resusc Emerg Med. 2022 Mar 24;30:23. doi: 10.1186/s13049-021-00985-0 (PMC8944068; doi:10.1186/s13049-021-00985-0)
Supplement: Supplementary file 2 — Additional file 2: Questionnaire on ultrasound and transmission quality [file 13049_2021_985_MOESM2_ESM.docx]

**Suppl 2: Remote Real-Time Supervision of Prehospital Point-of-Care Ultrasound: A Feasibility Study**

**Questionnaire**

| **Performed Ultrasound Scans** | **Yes** | **No** |
| --- | --- | --- |
| **Parasternal long** |  |  |
| **Parasternal short** |  |  |
| **Apical four-chamber** |  |  |
| **Subxiphoidal** |  |  |
| **Lung ultrasound** |  |  |
| **Morrison** |  |  |
| **Koller** |  |  |
| **Bladder** |  |  |

| **Tele-Supervision** | | |
| --- | --- | --- |
| **Quality of live stream** | 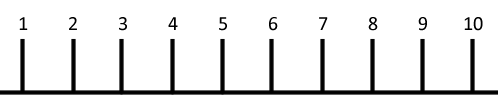  excellent poor | |
| **Quality of communication** | 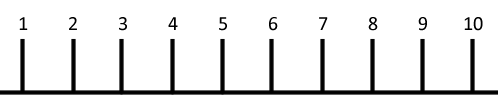  excellent poor | |
| **Expert available** | Yes  | No  |
| **Technical problems**  **If yes, specify** | Yes  | No  |
|  |  | |
| **Duration of ultrasound** | minutes | |
| **Duration of Tele-Supervision** | minutes | |
